# Investigation on Plugging and Profile Control of Polymer Microspheres as a Displacement Fluid in Enhanced Oil Recovery

**DOI:** 10.3390/polym11121993

**Published:** 2019-12-02

**Authors:** Xiangrong Nie, Junbin Chen, Yi Cao, Jinyuan Zhang, Wenjing Zhao, Yanlong He, Yunyi Hou, Shaomin Yuan

**Affiliations:** 1 College of Petroleum Engineering, Xi’an Shiyou University, Xi’an 710065, China; chenjbxu@126.com (J.C.); caoyi3891@xsyu.edu.cn (Y.C.); 15339079834@163.com (J.Z.); wenjing5767@163.com (W.Z.); stpnet@126.com (Y.H.); 2 Shaanxi Key Laboratory of Well Stability and Fluid Rock Mechanics in Oil and Gas Reservoirs, Xi’an Shiyou University, Xi’an 710065, China; 3 Research Institute of Shannxi Yanchang Petroleum (Group) Co. Ltd., Xi’an 710075, China; hyyfantasy@hotmail.com; 4 Exploration and Development Research Institute of Daqing Oil Field; Daqing 163712, China; smyuan@sina.com.cn

**Keywords:** polymer microspheres, profile control, enhanced oil recovery, pore throat, plugging

## Abstract

Polymer microspheres (PMs) are used as a new material to recover residual oil left in unswept oil areas after secondary recovery methods. The fact that the PMs plug the macropores causes the flow direction of the injection fluid to be transferred from macropores to micropores. In order to investigate the plugging and profile control mechanisms of PMs in reservoirs, four kinds of PMs with different particle sizes and four kinds of artificial cores with different permeability were selected for flooding tests, including plugging experiments and profile control experiments. The pore throat size distribution of cores was characterized by nuclear magnetic resonance (NMR) technology. The particle size distribution of PMs used in the experiment was characterized using a laser particle size analyzer. The results showed that there are six matching relationships existing simultaneously between pore throats and PMs based on theoretical analysis, which are completely plugging, single plugging, bridge plugging, smooth passing, deposition, and deformable passing. A key principle for optimizing PMs in profile control is that the particle size of the selected PMs can enter the high permeability layer well, but it is difficult for it to enter the low permeability layer. The results of this paper provide a theoretical basis for the optimal particle size of PMs during the oil field profile control process.

## 1. Introduction

Once the oil field is in the high water-cut stage, oil–water distribution in the reservoir becomes complicated, and reservoir heterogeneity becomes severe, causing water to bypass small pores and low-permeability layers, flowing along the large pores and high-permeability layers. As a result, the remaining oil in the small pores and the low-permeability strips cannot be displaced, forming an ineffective water circulation [1,2]. In order to further enhance oil recovery (EOR), it is possible to increase the sweep factor and oil displacement efficiency of the injected fluid. Profile control technology expands the sweep factor of the injected fluid by plugging the high permeability layer [3,4,5]. However, the traditional profile control agent only works in the near-well zone, and the subsequent injection of water quickly bypasses the plugged area and re-enters the high-permeability layer [6]. Therefore, a variety of in-depth profile control and flooding (PCF) techniques were proposed [7,8,9], including weak gels [10], colloidal dispersion gels [11], alkaline soil [12], foam [13], microbial [14], and oily sludge [15]. In order to further improve the in-depth PCF effect of the reservoir, scholars have proposed the idea of using polymer microspheres (PMs) for in-deep PCF.

The PMs are characterized by easy injection, good plugging, easy migration, and no damage to the reservoir. The PMs have small initial particle size and high dispersibility and are swelled in water after expansion in the reservoir [16,17,18,19]. When the PMs pass through the reservoir rock throat, the throat is blocked by a single or multiple microsphere bridges, forcing the deep liquid flow to enter the hypotonic zone and filling the reservoir with higher oil saturation. At the same time, the microspheres have good elasticity, can break through the plugging, and continue to block the reservoir to aid the redistribution of the liquid flow and the modifier [20,21,22,23,24]. The matching the relationship between the size of PMs and pore throats has played a particularly significant role in profile control and oil displacement [25,26]. When the injected PMs have a relatively large particle size, they only block the oil layer near the water injection well. The injected water bypasses the PMs and enters the high permeability layer again from the hydraulically connected low permeability layer, which leads to a poor effect of deep profile control. When the particle size of the PMs is too small, it is difficult to block the high permeability layer. Therefore, many researchers have conducted studies on optimizing the particle size of the polymer microspheres to increase the efficiency of PMs flooding [27,28,29,30,31].

**Figure 1:**
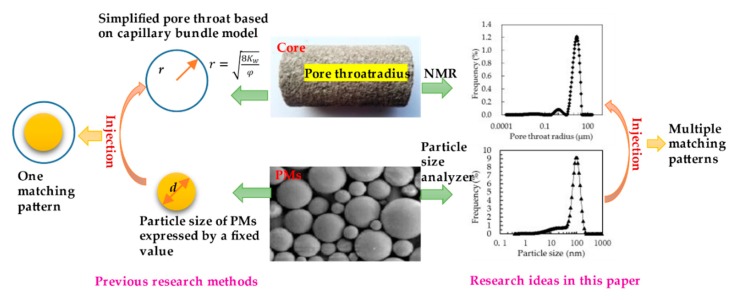
Sketch of previous research methods and research ideas in this paper.

As seen in Figure 1, previous studies have taken the pore throat size of the core and the particle size of PMs as fixed values [28,32]. In fact, the pore structure of the core is very complex, with different pore throat sizes [33,34,35]. Moreover, the size of PMs produced on a large scale by industrialization is not uniform [36,37]. Therefore, when PMs of different sizes are injected into cores with different pore sizes, multiple matching patterns will appear. Therefore, one of the purposes of this paper is to establish a more comprehensive matching mode between PMs and pore throats. In addition, the predecessors evaluated the profile control ability of PMs through a large number of physical simulation experiments and obtained some qualitative knowledge [38,39,40,41]. However, there is a lack of detailed description of the intrinsic mechanism of profile control performance of PMs. Therefore, the second purpose of this paper is to describe in detail the internal mechanism of polymer microspheres’ profile control from a mesoscopic perspective. In this paper, we conducted core flooding experiments and theoretical analysis to achieve the two aims. First, the physical properties of PMs with four particle sizes were characterized. Then, the pore throat distribution of core samples used for flooding experiments was characterized by NMR. In addition, flooding experiments were conducted, including the blocking rate experiments and profile control experiments. In the end, we comprehensively analyze the above experiments to reveal the plugging and profile control mechanisms.

## 2. Experimental Section

### 2.1. Materials

The properties of simulated formation water (SFW) used in this paper are indicated in Table 1. Before experimentation, the SFW is filtered through a membrane filter with 0.45 μm pore size.

**Figure 2:**
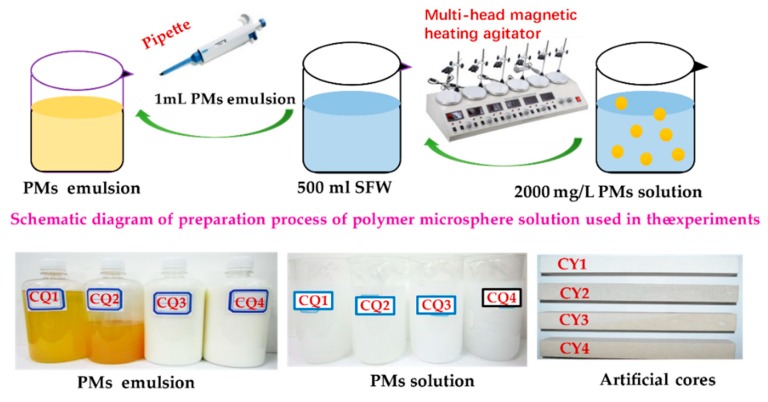
Schematic diagram of the preparation process of polymer microsphere solution and photographs of artificial cores and polymer microspheres used in the experiment.

The PMs used in the experiments were prepared by inverse emulsion polymerization and supplied by Xi’an Changqing Chemical Group Co., Ltd. The kind of the PMs is non-ionic, and the relative molecular weight is about 1.76 × 10^7^. The concentration of the PM solution used in the following experiments is 2000 mg/L. Four PMs with different particle diameter were used, including CQ1, CQ2, CQ3, and CQ4. In order to get the PM solution used in the following experiments, 1 mL PM emulsion was dissolved in 500 mL SFW using pipette to get 2000 mg/L PM solution, followed by stirred 10 min at 282 rpm until the microspheres evenly dispersed in the solvent using a multihead magnetic heating agitator. The macroscopic morphology of PM emulsion with a small particle size is a light-yellow liquid, and the large particle sizes are a milky white liquid. The formed PM solutions were all white liquids (Figure 2).

The artificial cores made of epoxy resin bonded with quartz sand were used in the experiments supplied by China University of Petroleum (Beijing) (Figure 2), which were made into rectangular cores (300 mm length × 45 mm width × 45 mm height) to be used in flooding experiments. Four kinds of artificial cores with different physical properties are displayed in Table 2.

### 2.2. Characterizations

The morphology of the PM solution was obtained with the assistance of an optical microscope (LEICA-DMLP, Germany Leica Corporation, Wiesner, Germany). A laser particle size analyzer (90Plus, Brookhaven Instruments Corporation, New York, USA) was used to determine the particle size distribution of the PMs. The pore throat radius distribution of the core samples was analyzed by a nuclear magnetic resonance (MiniMR-HTHP, Shanghai Niumag Analytical Instrument Co., Ltd., Shanghai, China).

### 2.3. Core Flooding Experiments

#### 2.3.1. Plugging Abilities of PMs

**Figure 3:**
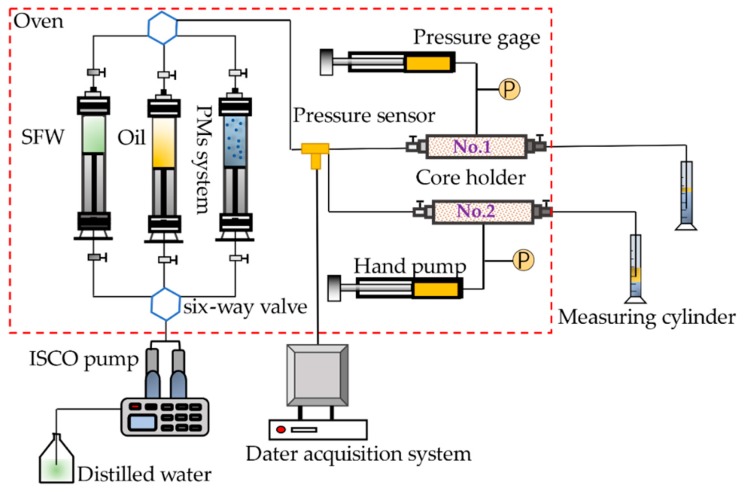
Schematic diagram of the core flooding apparatus.

A core flooding apparatus was used to evaluate the plugging abilities and profile control of PMs. The schematic diagram of the experimental setup is shown in Figure 3. All the flooding experiments in this paper were conducted at 25 °C. The fluid injection rate of all the flooding experiments was constant at 0.5 mL/min. A single core hold (No.1) was used in the experiments. The procedures were as follows: (1) Vacuum and saturate the core sample with brine, and then measure the pore volume; (2) the experimental instruments were set up as shown in Figure 3; (3) high-purity N_2_ was injected into the system to ensure it was properly sealed; (4) the core sample was mounted in the core holder, and the absolute permeability was determined by injecting SFW; (5) 0.2 PV (pore volume) of the PMs system was injected into the core sample; (6) then, the SFW was injected again until the pressure remained constant.

#### 2.3.2. Profile Control of PMs

Double core holds (No.1 and No.2) were used in the experiments. The specific procedures were as follows: (1) Inject 1 PV of SWF, and record the partial flow of the high and low permeability cores; (2) then, inject 1 PV of PMs system, and record the partial flow of the high and low permeability cores; (3) the SFW was injected again with an injection volume of 1.0 PV, and the partial flow of the high and low permeability cores was recorded.

## 3. Results

### 3.1. Physical Properties of the PMs

**Figure 4:**
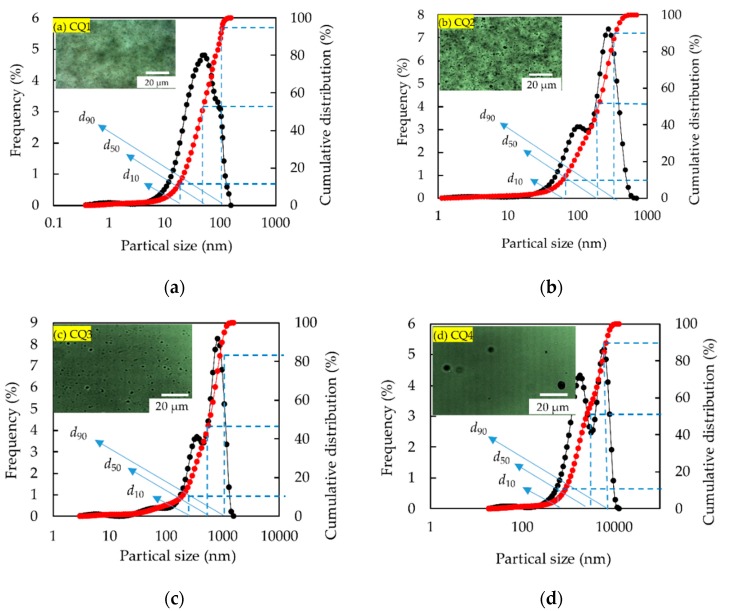
Morphology and particle size distribution of the PMs: (**a**) CQ1, (**b**) CQ2, (**c**) CQ3, and (**d**) CQ4.

**Figure 5:**
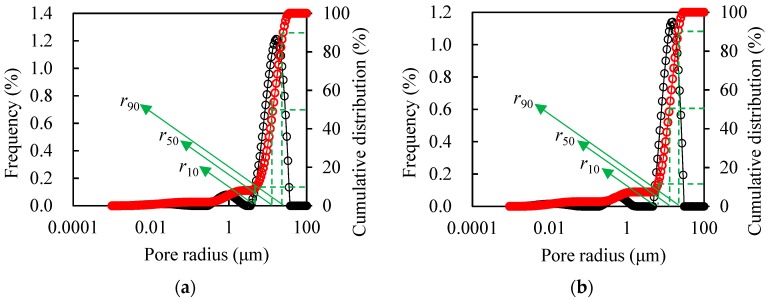

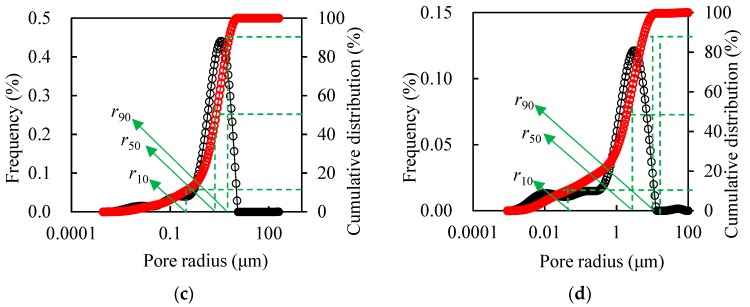
Pore throat distribution of cores: (**a**) CY1, (**b**) CY2, (**c**) CY3, and (**d**) CY4.

As shown in Figure 4, the PM solution is micrograde spherical in appearance and has good sphericity. Figure 4a shows that the particle size distribution of CQ1 exhibits a single peak morphology, indicating that the particle size is relatively uniform. Figure 5b,c shows that the particle size distribution of CQ2 and CO3 exhibit inconspicuous bimodal morphology, indicating that the particle size is not very uniform. Figure 5d shows that the particle size distribution of CQ4 shows a distinct bimodal morphology, indicating that the uniformity of the particle size is worse than CQ2 and CQ3. The characteristic value of particle size cumulative distribution curve of PMs is shown in Table 3, where *d*_10_, *d*_50_, and *d*_90_ refer to the particle size when the cumulative distribution of microspheres reaches 10%, 50%, and 90%.

### 3.2. Pore Throat Distribution of Cores

Figure 5a–d illustrates the pore throat distribution of CY1, CY2, CY3, and CY4, exhibiting a single peak morphology, indicating that the pore throat of the artificial cores is relatively uniform. The characteristic value of pore radius cumulative distribution curve of cores is shown in Table 4, where *r*_10_, *r*_50_, and *r*_90_ refer to the pore size when the cumulative distribution of cores reaches 10%, 50%, and 90%.

### 3.3. Plugging Abilities of PMs

**Figure 6:**
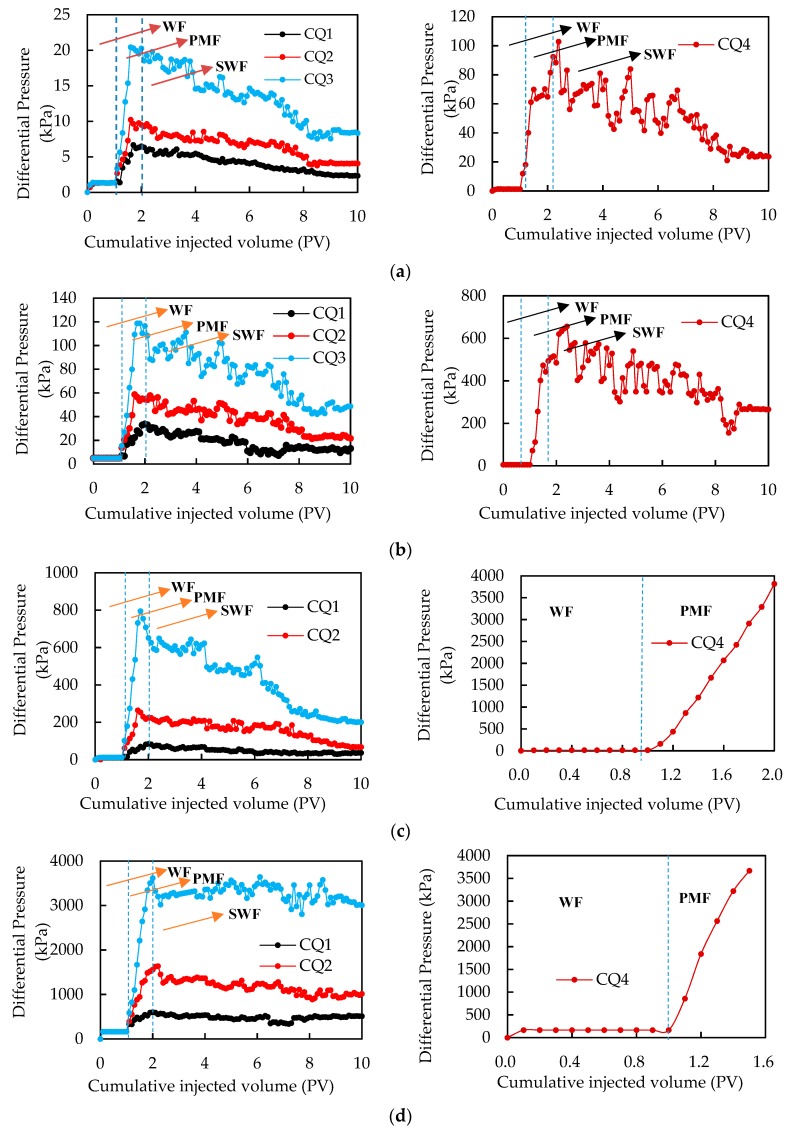
Pressure differential curves of PMs injected into cores: (**a**) CY1, (**b**) CY2, (**c**) CY3, and (**d**) CY4.

Core flooding experiments were carried out to evaluate the plugging abilities of PMs in artificial cores. The results are shown in Figure 6. The injection process can be divided into three injection stages: water flooding (WF) stage, PM flooding (PMF) stage, and finally, subsequent water flooding (SWF) stage after injection of a slug of PMs. The CQ4 PMs were injected into the CY3 and CY4 core samples, and the pressure increased sharply beyond the preset pressure, thus stopping the injection (Figure 6c,d). In the WF stage, the differential pressure kept constant. Then, in the PMF stage, the differential pressure sharply increased and then fluctuated with an approximately 1 PV PM system being injected into core samples. The reason for this test phenomenon is that when the PMs block the pore throat, the pressure rises to a maximum value, and then as the pressure difference increases, the polymer microspheres successfully pass through the pore throat, causing the pressure to drop. Then, when the next blockage occurs, the pressure difference continues to rise, and this process repeats, again and again, causing pressure fluctuations. At the SWF stage, the pressure begins to decrease gradually. The more the water is injected, the more the pressure difference decreases. The pressure difference after accumulative injection of 8PV SFW is much higher than that at the initial stage of water flooding, which indicates that the PMs have good plugging performance. The results showed that the microspheres can plug the pore throat by adsorption, aggregation, and bridging.

### 3.4. Profile Control of PMs

In profile control with PMs, there are three typical cases: (1) PMs can easily be injected into a high permeable layer, but it is difficult for them to be injected into a low permeable layer; (2) PMs can easily inject into high permeable layer and low permeable layer equally; (3) PMs can hardly be injected into a low permeable layer and high permeable layer. In order to analyze the above cases by experimentation, we selected PMs and cores for profile control experiments based on the results shown in Figure 6. It can be seen from Figure 6b,c that CQ4 PMs can be easily injected into the CY2 core, but it is difficult for them to be injected into the CY3 core, so we used CQ4 PMs and CY2 and CY3 cores to simulate the first typical case. Similarly, it can be seen from Figure 6b,c that CQ1 PMs can be easily injected into CY2 and CY3 cores, so we used CQ1 PMs and CY2 and CY3 cores to simulate the second case. It can be seen from Figure 6c,d that it is difficult to inject CQ4 PMs into CY3 and CY4 cores, so we used CQ4 PMs and CY3 and CY4 cores to simulate the third case.

**Figure 7:**
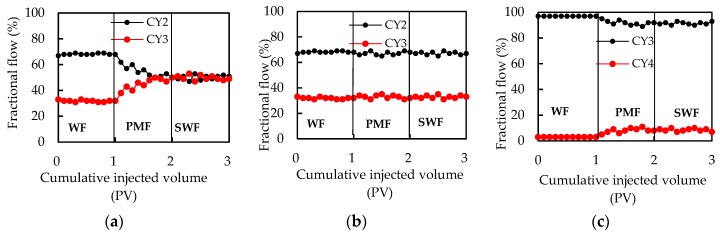
Fractional flow curves in the PM injection process: (**a**) CQ4, (**b**) CQ1, and (**c**) CQ4.

Fractional flow experiments were used to investigate the selective plugging and profile control mechanism of PMs. Figure 7a shows that in WF stage, the fractional flow of CY2 core and CY3 core is 67%, and 23%, respectively. During the PMF process, the fractional flow of CY3 core gradually increases, and the fractional flow of CY2 core gradually decreases. The fractional flow of CY2 core and CY3 core are both approximately 50% when the PM injection volume reaches 1PV. The results showed that the CQ4 PMs can effectively improve the injection profile of the heterogeneous cores. However, Figure 7b,c shows that there is almost no change in the fractional flow curves after injecting CQ1 PMs and CQ4 PMs, respectively. In other words, CQ1 PMs and CQ4 PMs could not play a role in profile control for the CY2 core and CY3 core, and the CY3 core and CY4 core, respectively. Through a comprehensive analysis of the above three experiments, it is shown that the PMs have selective plugging properties.

## 4. Discussion

### 4.1. Six Matching Patterns of PMs in Core

**Figure 8:**
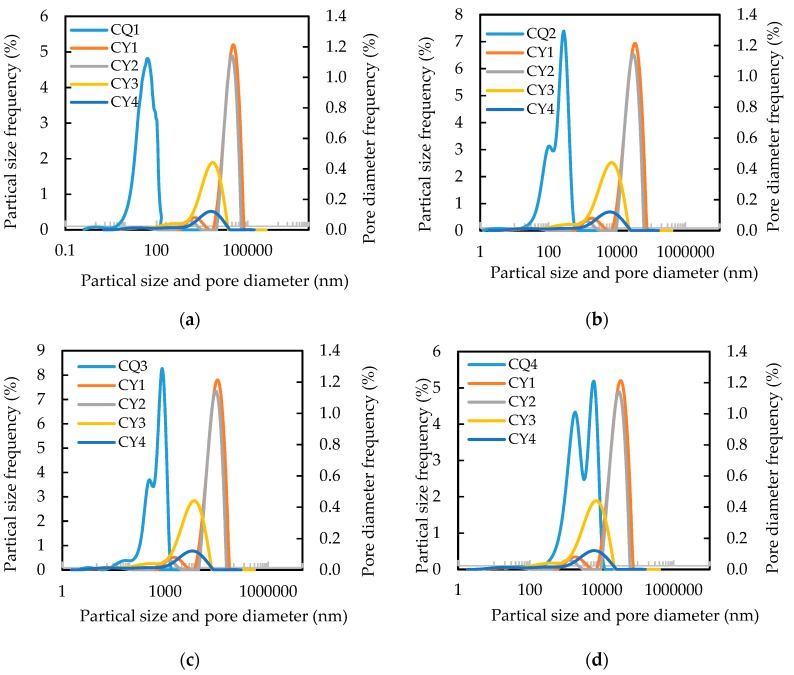
Diameter of pore throat and particle size of PM distribution curves: (**a**) CY1, CY2, CY3, CY4 and CQ1, (**b**) CY1, CY2, CY3, CY4 and CQ2, (**c**) CY1, CY2, CY3, CY4 and CQ3, (**d**) CY1, CY2, CY3, CY4 and CQ4.

In order to accurately characterize the matching relationship between polymer microspheres and pore throats, the pore throat radius distribution curves of four cores were transformed into pore throat diameter distribution curves, and the same diagrams were drawn with the particle size distribution curves of four polymer microspheres, as shown in Figure 8. The predecessor proposed a method for determining the matching pattern. At first, the average pore throat diameter of the core was calculated by Equation (1) [27]. Then, in combination with the size of the PMs, the matching pattern can be determined. As shown in Figure 8, the pore throat size of the core varies widely, rather than the value calculated by Equation (1). Further, the size of the PMs is not a constant value but varies within a certain range. Obviously, the method proposed by predecessors is very rough.

(1) $$D=2\times \sqrt{\frac{8K_{w}}{\varphi }}$$

where *D* is the average pore throat diameter of core, μm; *K_w_* is the core permeability after water flooding, μm^2^; and *φ* is the porosity of the core.

**Figure 9:**
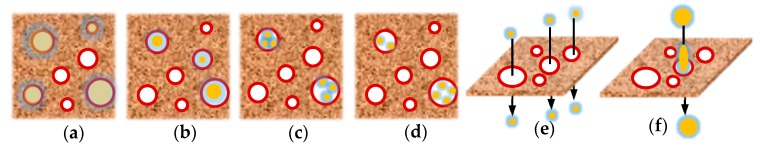
Six plugging types of PMs in the core: (**a**) Complete plugging, (**b**) Single plugging, (**c**) Bridge plugging, (**d**) Deposition, (**e**) Smooth passing, (**f**) Deformable passing.

The particle size distribution curve of CQ3 PMs and the pore throat diameter distribution curve of the CY3 core in Figure 8c were analyzed as examples. The two curves coincide partly, which indicates that the matching relationship between CQ3 PMs and pore throat is very complex. Through theoretical analysis, we summarize six typical matching models, as shown in Figure 9. Figure 9a shows that a PMs could not enter the pore since the PM size was much larger than that of the pore throat, which we name complete plugging. When the PMs and the pore throat have a close size, as shown in Figure 9b, one PM could plug the pore effectively, which we name single plugging. As pointed out in Figure 9c, some PMs formed aggregates to bridge the pore when the PMs were 2–3 times smaller than the pore, which we name bridge plugging. The PMs flow in a porous medium, they are subjected to various forces. When the resultant force is directed to the surface of the pores, the microspheres are deposited, which we name deposition, as shown in Figure 9d. The PMs could easily pass through the pores with barely any plugging, owing to their smaller size relative to the pore size, as shown in Figure 9e, which we name smooth pass. The PMs have certain deformability and can flow through the pore throat through deformation, which we name deformable passing, as shown in Figure 9f.

### 4.2. Plugging Abilities of PMs in Core

As shown in Figure 8a, the particle size distribution curve of CQ1 PMs hardly coincides with the pore throat diameter curves of the CY1 and CY2 cores, and coincides very little with the CY3 and CY4 cores. This indicates that CQ1 PMs have very weak plugging properties for CY1 and CY2 cores, and weak plugging properties for CY3 and CY4 cores. The overlap part of the curves in Figure 8b is slightly larger than that in Figure 8a, indicating that CQ2 PMs have a slightly better plugging performance than CQ1 PMs. The coincidence part of the curves in Figure 8b,c increases gradually, which indicates that CQ3 PMs and CQ4 PMs have a better plugging ability, but the coincidence area of the CY4 core and CQ3 PMs and CQ4 PMs is larger, which indicates that the two PMs may be difficult to inject into the CY4 core. The same results can be obtained from Figure 6. In this work, the resistance coefficient and blocking rate were used to quantitatively characterize the plugging ability, calculated by Equations (2) and (3). The results are shown in Table 5. For the same core, the larger the particle size of PMs, the higher the resistance coefficient and blocking rate, which means the better the plugging effect. However, when the particle size of PMs exceeds the pore size of the core, it is difficult for the PMs to be injected into the core.

(2) $$F_{r}=\frac{\lambda_{w}}{\lambda_{p}}=\frac{{\left(k/\mu \right)}_{w}}{{\left(k/\mu \right)}_{p}}=\frac{\Delta P_{p}}{\Delta P_{w}}\times \frac{Q_{p}}{Q_{w}}v$$

(3) $$\eta =1-\frac{K_{sw}}{K_{w}}$$

where *F_r_* is the resistance coefficient and η is the blocking rate; *K_w_*, *K_p_*, and *K_sw_* respectively represent the core permeability after WF, PMF, and SWF, μm^2^; *μ_w_* and *μ_p_* are, respectively, viscosity of water and PM system, mPa·s; Δ*P_w_* and Δ*P_p_* represent, respectively, the differential pressure of WF and PMF, MPa; and *Q_w_* and *Q_p_* are, respectively, the injection rate during WF and PMF, mL/min.

### 4.3. Migration Process of PMs in Core Sample

**Figure 10:**
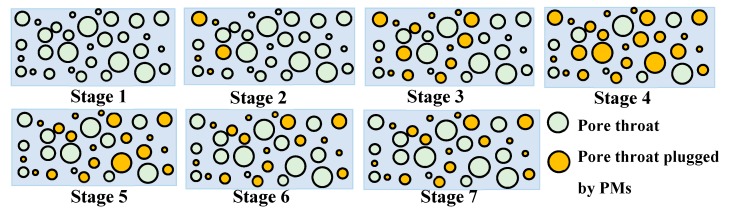
Typical migration diagrams of PMs in core.

Because the artificial core is opaque, it is difficult to observe the movement of PMs in the core directly. The typical migration process of PMs in the core can be obtained indirectly by analyzing the pressure difference curves shown in Figure 6. As shown in Figure 10, the first stage indicates that the pore throats of the core are saturated by SFW. The second stage shows that the PMs enter the core and mainly accumulate near the injection end. With the continuous injection of PMs, the third and fourth stage indicate that the PMs gradually migrate to the deep core, accompanied by more pore throats plugged by PMs. During subsequent water injection (stages 5, 6, and 7), some PMs flow out of the core, resulting in some blocked pore throats being dredged. With the continuous water injection, more and more PMs flow out of the core, but some of them remain in the core and play a role in profile control.

### 4.4. Profile Control Mechanisms

**Figure 11:**
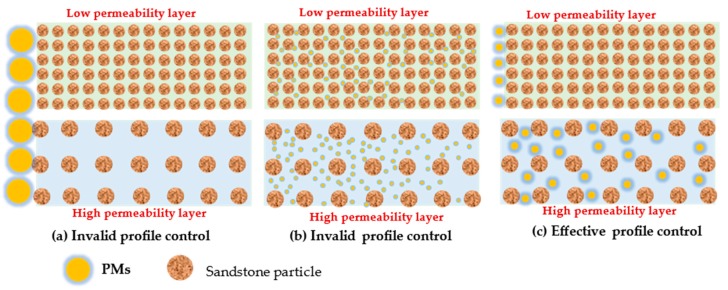
Schematic diagram of effective profile control and ineffective profile control.

Figure 7a shows that cq4 PMS can effectively plug the CY2 core, which results in the injection liquid transferring from the CY2 core to CY3 core, showing a good profile control effect. Figure 7b,c shows an invalid profile control. Combining with Figure 8, we can summarize the mechanisms of effective profile control and ineffective profile control. As shown in Figure 11a, when the particle diameter of the PMs is larger than the pore throat diameter of the high permeability layer, the PMs cannot be injected into the high permeability layer, and the profile control is invalid. As shown in Figure 11b, the PMs can enter the low permeability layer when the particle diameter of the PMs is smaller than the pore throat diameter of the low permeability layer, resulting in a decrease in the permeability of the low permeability layer, and invalid profile control. Profile control is effective only when the polymer microspheres are able to enter the high permeability layer and are unable to enter the low permeability layer, as shown in Figure 11c. That is to say, the particle diameter of the PMs is smaller than the pore throat diameter of the high permeability layer, but larger than the pore throat diameter of the low permeability layer.

## 5. Conclusions

In this work, we focused on plugging and profile control of PMs in porous media using physical experiments. The following conclusions are made:

- The particle size distribution of PMs was measured by a laser particle size analyzer, and the pore size distribution of the core was measured by NMR. The above results show that the size of PMs and the pore varies in a certain scale, which indicates that the previous studies were not accurate based on the average pore throat size and fixed particle size. Therefore, we point out that the particle size distribution and pore throat distribution should be fully considered in future research about plugging and migration.
- There is an overlap between the particle size distribution curve of PMs and the pore size distribution curve of the core in the same coordinate system, which shows some of the small microspheres can easily flow through the pore throat, and other of the big microspheres can hardly flow through the pore throat, resulting in various matching patterns. Based on theoretical analysis, we summarize six typical plugging types, which are completely plugging, single plugging, bridge plugging, smooth passing, deposition, and deformable passing.
- Through the plugging ability experiments, we found that plugging ability is related to the overlapping area of the PMs’ particle size distribution curve and the pore throat diameter distribution curve. The larger the overlapping area of the two curves, the better the plugging ability. However, it is difficult to establish the quantitative relationship between the overlapping area of the two curves and the plugging ability.
- The pressure difference decreased in the SWF stage, indicating that some PMs flowed out from the end face of the core. The typical migration stages of PMs in the core are the stage of microspheres flowing into the core, the stage of microspheres deep migration, and the stage of some microspheres flowing out of the core.
- When PMs are used for profile control, the particle size of PMs must meet certain conditions. Specifically, it is necessary to ensure that PMs can enter the high permeability layer, but not the low permeability layer.

## Figures and Tables

**Table 1:** Properties of simulated formation water.

pH	Cation (mg/L)						Anion (mg/L)		Total Salinity(mg/L)	Water Type
	K^+^	Na^+^	Ca^2+^	Mg^2+^	Ba^2+^	Sr^2+^	HCO^3−^	Cl^−^		
7.31	2643	2711	241	42	55	61	313	8641	14707	CaCl_2_

**Table 2:** Petrophysical properties of core samples.

Sample No.	CY1	CY2	CY3	CY4
Porosity (%)	28.87	23.08	15.81	5.11
Permeability (10^−3^ μm^2^)	1387.04	769.86	354.73	10.29

**Table 3:** Characteristic value of particle size cumulative distribution curve of polymer microspheres (PMs).

PMs No.	*d*_10_ (nm)	*d*_50_ (nm)	*d*_90_ (nm)	PMs No.	*d*_10_ (nm)	*d*_50_ (nm)	*d*_90_ (nm)
CQ1	17.183	47.939	104.748	CQ3	181.852	611.402	974.696
CQ2	62.121	208.857	365.511	CQ4	859.160	2631.32	6687.423

**Table 4:** Characteristic value of pore radius cumulative distribution curve of cores.

Core No.	*r*_10_ (μm)	*r*_50_ (μm)	*r*_90_ (μm)	Core No.	*r*_10_ (μm)	*r*_50_ (μm)	*r*_90_ (μm)
CY1	5.410	13.339	24.917	CY3	0.255	2.520	6.216
CY2	6.215	12.445	21.687	CY4	0.042	2.047	6.216

**Table 5:** Evaluation of blocking abilities of PMs.

Core No.	Resistance Coefficient				Blocking Rate (%)			
	CQ1	CQ2	CQ3	CQ4	CQ1	CQ2	CQ3	CQ4
CY1	1.74	3.05	6.26	17.75	42.67	67.16	84.01	94.37
CY2	2.70	4.51	10.12	55.14	63.03	77.83	90.12	98.19
CY3	2.99	5.53	16.42	-	66.52	81.91	93.91	-
CY4	3.10	6.16	18.22	-	67.78	83.76	94.51	-
